# Complete chloroplast genome of green alga *Caulerpa serrulata* (Forsskål) J.Agardh, 1837

**DOI:** 10.1080/23802359.2019.1636726

**Published:** 2019-07-12

**Authors:** Wei Tan, Rongxia Wang, Hongtao Liu, Yongbo Wang, Hongji Ke, Jiawei Fan, Fuxiao Chen

**Affiliations:** Hainan Provincial Engineering Research Center for Tropical Sea-farming, Hainan Academy of Ocean and Fisheries Sciences, Haikou, China

**Keywords:** Chloroplast genome, *Caulerpa serrulata*, phylogenetic tree

## Abstract

The complete chloroplast genome of *Caulerpa serrulata* from South China Sea has been assembled and characterized for the first time. The circular chloroplast genome was 177,848 bp in length, with a GC content of 33.80%. It contained 117 genes, which included 78 protein-coding genes, 37 tRNA genes, and 2 ribosomal RNA genes. Like other species in *Caulerpa*, the chloroplast genome of *C. serrulata* did not demonstrate a typical quadripartite structure. A total of 35 microsatellites (SSRs) were identified in the genome using MISA. Phylogenetic analysis showed that *C. serrulata* was closer to *Caulerpa cupressoides*, which further clarified the phylogenetic relationships of species in *Caulerpa*.

*Caulerpa serrulata*, commonly known as cactus tree alga or serrated green seaweed, is a species of seaweed in the family Caulerpaceae. It has a green to grey-green thallus that typically grows outward to around 30 centimeters. *Caulerpa serrulata* can be found in intertidal and subtidal zones in tropical waters around the world. It is edible and used as medicines to lower blood pressure and an antibacterial and antifungal agent. The secondary metabolites from the *Caulerpa* were also reported to have various biotechnological and pharmacological applications (Cavas and Pohnert 2010). For example, a new bis-indole, caulersin, was isolated from the alga *C. serrulata* (Su et al. 1997). In addition, after modification with ethylenediamine (EDA), the alga *C. serrulata* have a great potential for the removal of metals and dissolved organic carbon (Mwangi and Ngila 2012). A study recently showed that silver nanoparticles were eco-friendly synthesized using *C. serrulata* aqueous extract, which have a good catalytic and antibacterial activities (Aboelfetoh et al. 2017).

The fresh thalli of *C. serrulata* were collected from Yinggehai (N18°30′15.43″, E108°41′24.79″) in Ledong county, Hainan province, China. The voucher specimens were stored in Qionghai research base of Hainan Academy of Ocean and Fisheries Sciences. The samples were used for the total genomic DNA extraction with the modified CTAB method (Doyle 1987). The complete genome sequencing was conducted with 150 bp pair-end reads on the Illumina Hiseq Platform and assembled with NOVOPlasty (Dierckxsens et al. 2016). The annotations of chloroplast (cp) genome were submitted to GenBank database (Accession No. MK792749). The phylogenetic analysis was carried out based on super matrix of 21 PCGs in 9 cp genomes of species in *Caulerpa*, using maximum likelihood (ML) method with 1000 bootstrap replicates.

The complete cp genome of *C. serrulata* was 177,848 bp in length, the base content of A, T, G, and C was 32.18, 34.02, 16.97 and 16.83%, respectively, with the overall GC content of 33.80%. The whole cp genome contained 117 genes, including 78 protein-coding genes, 37 transfer RNA (tRNA) genes and 2 ribosomal RNA (rRNA) genes. Like other species in *Caulerpa*, the cp genome of *C. serrulata* did not demonstrate a quadripartite structure and lacked the large rRNA operon-encoding inverted repeat (IR)(Yan et al. 2018). Forty-five PCGs and 28 tRNA genes were encoded on the forward strand and 33 PCGs, 9 tRNA genes, and 2 rRNA genes were encoded on the reverse strand. In addition, a total of 35 microsatellites (SSRs) were identified in the *C. serrulata* cp genome using MISA. Among these SSRs, 33 were mononucleotides (A/T), one was dinucleotide (AT), and one was hexanucleotide (TGGAAG).

The results of the phylogenetic tree (Figure 1) revealed that *C. serrulata* was closer to *Caulerpa cupressoides* which was first clustered with *Caulerpa manorensis*, *Caulerpa okamurae*, and *Caulerpa racemose*. This result is basically consistent with the previous work (Yeh and Chen 2004; Kazi et al. 2013). The data of *C. serrulata* cp genome will be useful for further studies on phylogeny and evolution of green alga Bryopsidales.

**Figure 1.**
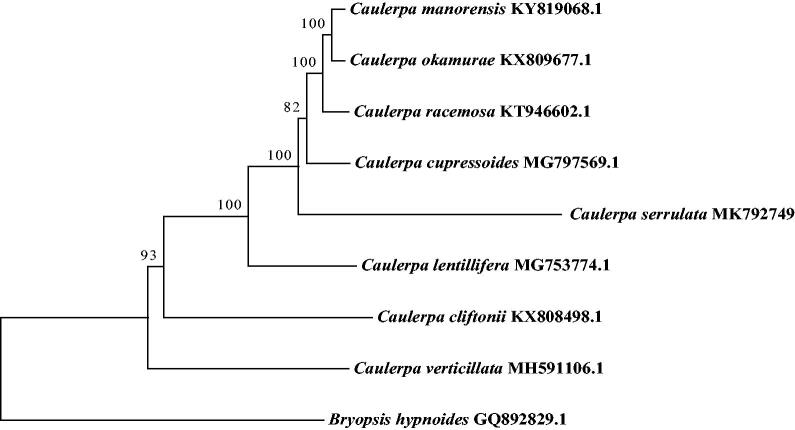
Phylogenetic tree of 9 species based on 21-PCGs from chloroplast genome by maximum likelihood (ML) method.

*Bryopsis hypnoides* was used as an outgroup.
